# *Poria cocos* Attenuates LPS/D-Galactosamine-Induced Acute Liver Failure in Rats: An Integrative Exploratory Study Combining Network Pharmacology and In Vivo Validation

**DOI:** 10.3390/ijms27031403

**Published:** 2026-01-30

**Authors:** Peihua Wen, Xinru Jian, Xiaoyu Ren, Shusen Zhao, Yuhan Yang, Haotian Ge, Longjie Li, Hongxun Wang, Maoteng Li, Limei Wang

**Affiliations:** 1 School of Life Science and Technology, Wuhan Polytechnic University, Wuhan 430048, Chinalilongjie@whpu.edu.cn (L.L.);; 2 Xianghu Laboratory, Biological Seed Industry Research Institute, Hangzhou 311231, China; 3 Key Laboratory of Molecular Biophysics of the Ministry of Education, College of Life Science and Technology, Huazhong University of Science and Technology, Wuhan 430074, China

**Keywords:** acute liver failure, *Poria cocos*, network pharmacology, PI3K/AKT pathway, inflammation

## Abstract

Acute liver failure (ALF) is a rapidly progressive and life-threatening condition with limited pharmacological interventions. *Poria cocos*, a medicinal fungus widely used in traditional Chinese medicine, has been reported to exhibit anti-inflammatory and hepatoprotective activities; however, its potential involvement in ALF remains incompletely understood. In this study, an integrative exploratory strategy combining network pharmacology, molecular docking, and in vivo experiments was employed to investigate the protective effects of *Poria cocos* against lipopolysaccharide/D-galactosamine (LPS/D-GalN)-induced ALF in rats. Rats were pretreated with *Poria cocos* extract (50 or 200 mg/kg), and hepatoprotective effects were assessed by survival analysis, serum biochemical indicators(alanine aminotransferase [ALT], aspartate aminotransferase [AST], total bilirubin [TBil], and international normalized ratio [INR]), histopathology, and expression of inflammation- and PI3K/AKT-related markers. Network pharmacology analysis identified fifteen putative bioactive components of *Poria cocos* and 178 ALF-related overlapping targets, with enrichment analyses highlighting multiple inflammation-, apoptosis-, and PI3K/AKT-related signaling pathways. Molecular docking suggested potential interactions between major components and predicted core targets. In vivo, *Poria cocos* pretreatment was associated with improved survival, alleviated liver injury, and reduced the expression of inflammatory and apoptosis-associated markers, including PI3K, AKT1, NF-κB, TNF-α, MAPK14(p38), Caspase-3, and MMP2. Taken together, network pharmacology analysis identified PI3K/AKT-associated signaling as a candidate pathway, and the in vivo findings were generally consistent with this prediction, suggesting that the hepatoprotective effects of *Poria cocos* may involve multi-target regulation of inflammation- and apoptosis-related pathways.

## 1. Introduction

Acute liver failure (ALF) is a rapidly progressive and life-threatening clinical syndrome marked by extensive hepatocellular necrosis, which leads to acute hepatic dysfunction, hepatic encephalopathy, and coagulation abnormalities [1]. The etiology of ALF is heterogeneous and includes drug-induced liver injury, particularly acetaminophen overdose [2], exposure to food-borne or environmental toxins [3], and autoimmune liver diseases [4]. At the pathophysiological level, ALF is associated with oxidative stress [5], mitochondrial dysfunction [6], dysregulated hepatocyte death, including apoptosis and necrosis [7,8], and an excessive systemic inflammatory response [9]. These interconnected processes interact and collectively contribute to the rapid deterioration of liver function.

Although progress has been made in understanding the pathogenesis of ALF, clinical management remains limited. Current treatment strategies mainly rely on intensive supportive care, while liver transplantation remains the only definitive therapeutic option; however, its application is restricted by donor availability, surgical risk, and economic constraints [10]. Pharmacological strategies capable of effectively preventing or slowing ALF progression are still lacking. Consequently, identifying preventive approaches that target early liver injury, particularly in high-risk settings, remains an important clinical objective.

Traditional Chinese medicine (TCM) has long been utilized in the prevention and management of liver diseases and offers a rich source of bioactive compounds with multi-target regulatory characteristics [11,12]. *Poria cocos*, a medicinal fungus widely distributed in China and Southeast Asia [13], contains polysaccharides, triterpenoids, sterols, and other secondary metabolites [14,15]. Previous studies have shown that *Poria cocos* and its constituents possess anti-inflammatory, antioxidant, and immunomodulatory properties [16], and may also influence metabolic regulation and gut microbiota composition [17,18]. While the protective effects of *Poria cocos* extracts have been reported in several experimental models of liver injury [19,20], their preventive effects during the early phase preceding acute liver failure have not been systematically investigated. In particular, evidence derived from the lipopolysaccharide/D-galactosamine (LPS/D-GalN)-induced ALF model remains limited.

Network pharmacology provides a systems-oriented framework for investigating the multi-component and multi-target characteristics of herbal medicines [21]. By integrating chemical data with target prediction and disease-related databases, this approach enables the construction of compound–target–pathway networks that can guide experimental exploration [22]. Enrichment analyses based on Gene Ontology (GO) and the Kyoto Encyclopedia of Genes and Genomes (KEGG) are commonly used to identify biological processes and signaling pathways potentially involved in disease progression [23]. However, predictions generated by network pharmacology and molecular docking are inherently theoretical and should be interpreted cautiously in conjunction with experimental observations.

In this study, network pharmacology analysis, molecular docking, and in vivo experiments were combined to explore the potential preventive effects of *Poria cocos* in ALF. Major bioactive compounds were screened, and their putative targets were predicted to construct a compound–target–pathway network. Molecular docking was subsequently applied to assess the theoretical binding feasibility between representative compounds and selected core targets. An LPS/ D-GalN-induced rat model of ALF was then established, in which *Poria cocos* extract was administered for seven consecutive days prior to model induction. Preventive effects were evaluated based on liver function parameters, histopathological alterations, inflammatory markers, and apoptosis-related molecular expression. Through this integrative approach, this work seeks to provide experimental support for the preventive potential of *Poria cocos* and to offer exploratory insight into herbal-based strategies for liver injury prevention. Accordingly, the present study was designed as an exploratory, hypothesis-generating investigation and was not intended to establish pathway dependency or causal molecular mechanisms.

## 2. Results

### 2.1. Network Pharmacology and Molecular Docking

#### 2.1.1. Identification of Active Components, Predicted Targets, and Network Construction

A multi-database integration and cross-validation strategy was applied to predict potential targets of *Poria cocos*. Active compounds were screened using predefined criteria of oral bioavailability (OB) ≥ 30% and drug-likeness (DL) ≥ 0.18, which are commonly adopted thresholds in Traditional Chinese Medicine Systems Pharmacology Database and Analysis Platform (TCMSP)-based network pharmacology studies (Table 1). In addition, Liquid Chromatography-Mass Spectrometry (LC–MS) analysis was performed to provide qualitative compositional characterization of the *Poria cocos* extract, and the results are presented in the Supplementary Materials (Figure S5). By integrating target prediction results from TCMSP and the SwissTargetPrediction database, 309 potential targets were preliminarily identified. The PharmMapper server and the Similarity Ensemble Approach (SEA) database were also used for independent target prediction. After integrating results from all databases (TCMSP, SwissTargetPrediction, PharmMapper, and SEA), the final set of potential targets associated with *Poria cocos* was obtained (Figure S1).

**Figure 1:**
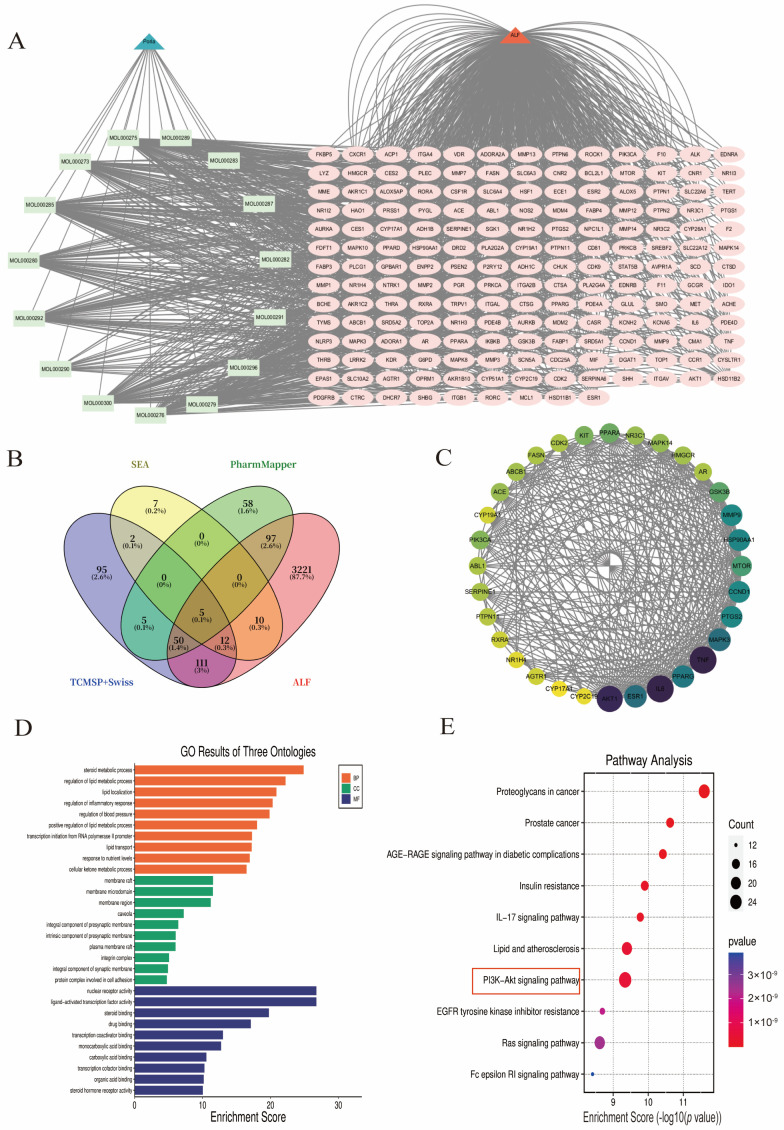
Network-based analysis of *Poria cocos* in acute liver failure (ALF). (**A**) Protein–protein interaction (PPI) network constructed based on overlapping targets between *Poria cocos* bioactive compounds and ALF-related targets. (**B**) Venn diagram showing 178 overlapping targets between *Poria cocos*-related targets and ALF-associated targets. (**C**) Identification of 32 core targets based on network topological parameters, including degree, closeness centrality, and betweenness centrality (thresholds set at the corresponding median values). (**D**) Gene Ontology (GO) enrichment analysis of the overlapping targets, showing the top 10 enriched terms for molecular function (MF), cellular component (CC), and biological process (BP). (**E**) Kyoto Encyclopedia of Genes and Genomes (KEGG) pathway enrichment analysis showing the top 10 significantly enriched signaling pathways. The pathway marked by the red box exhibited both the smallest *p*-value and the largest count, identified as the PI3K/AKT signaling pathway (hsa04151), with 11 candidate targets, an enrichment ratio of 35.48, and a *p*-value of 1.92 × 10^−7^.

A total of 3378 targets were screened from the GeneCards database based on a relevance score > 1; 283 targets were obtained from the DisGeNET database with a disease association score > 0.01; 359 targets directly associated with ALF were collected from the OMIM database; and 2 therapeutically annotated ALF-related targets were retrieved from the TTD database (Figure S2). After merging datasets and removing duplicates, a total of 3507 ALF-related targets were identified, and all target identifiers were standardized to official gene symbols using the UniProt database. Intersection analysis identified 178 overlapping targets between *Poria cocos*-related targets and ALF-related targets, which were visualized using a Venn diagram (Figure 1B). These overlapping targets were imported into the STRING database to construct a protein–protein interaction (PPI) network and visualized using Cytoscape (Version 3.9.1). The resulting PPI network contained 178 nodes and 1907 edges, with an average node degree of 21.5 (Figure S3). A *Poria cocos*–ALF interaction network was constructed (Figure 1A) and used for subsequent core target screening and pathway enrichment analysis.

#### 2.1.2. Core Target Screening and GO/KEGG Enrichment Analysis

Network topological analysis of the PPI network was conducted using the CytoNCA plugin in Cytoscape 3.9.1 to identify core targets. The assessment was based on three topological parameters: degree, closeness centrality, and betweenness centrality. The average values of these parameters were 21.548, 0.0027, and 200.079, respectively. Based on this multi-parameter evaluation, a total of 32 core targets were identified. These targets showed high topological relevance within the interaction network. A refined PPI network was reconstructed using these core targets, containing 32 nodes and 304 edges (Figure 1C).

GO enrichment analysis revealed 2350 significant biological process (BP), 103 cellular component (CC), and 210 molecular function (MF) terms (*p* < 0.01, FDR < 0.05), with the top 10 enriched terms shown in Figure 1D. The enriched GO terms were primarily associated with inflammation-related processes, regulation of gene expression, and signal transduction-related functions. KEGG pathway enrichment analysis further revealed several significantly enriched pathways, among which the PI3K/AKT signaling pathway (hsa04151; 11 candidate targets, enrichment ratio = 35.48, *p* = 1.92 × 10^−7^) was identified (Figure 1E).

#### 2.1.3. Molecular Docking of Active Components with Core Proteins

**Figure 2:**
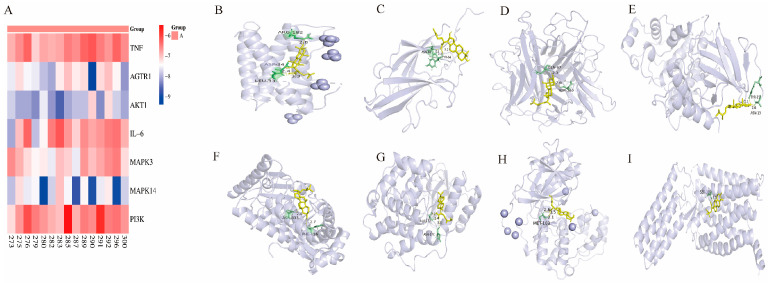
(**A**) Predicted binding energies of representative *Poria cocos* bioactive compounds with key target proteins predicted by network pharmacology analysis; (**B**) Predicted docking conformation of IL-6 with (2R)-2-[(3S,5R,10S,13R,14R,16R,17R)-3,16-dihydroxy-4,4,10,13,14-pentamethyl-2,3,5,6,12,15,16,17-octahydro-1H-cyclopenta[a]phenanthren-17-yl]-6-methylhept-5-enoic acid; (**C**) Predicted docking conformation of PI3K with (2R)-2-[(3S,5R,10S,13R,14R,16R,17R)-3,16-dihydroxy-4,4,10,13,14-pentamethyl-2,3,5,6,12,15,16,17-octahydro-1H-cyclopenta[a]phenanthren-17-yl]-6-methylhept-5-enoic acid; (**D**) Predicted docking conformation TNF-α with cerevisterol.; (**E**) Predicted docking conformation of MAPK3 with ergosta-7,22E-dien-3β-ol; (**F**) Predicted docking conformation of AKT1 with ergosterol peroxide; (**G**) Predicted docking conformation of MAPK3 with 3β-hydroxy-24-methylene-8-lanostene-21-oic acid; (**H**) Predicted docking conformation of MAPK14 (p38) with 3β-hydroxy-24-methylene-8-lanostene-21-oic acid; (**I**) Docking model of AGTR1 with dehydroeburicoic acid. In the docking conformations (**B**–**I**), amino acid residues are shown in green, ligand molecules in yellow, and hydrogen bonds are indicated by dashed lines.

Molecular docking analysis using AutoDock Vina (Version 1.1.2) showed binding energies below −6.0 kcal/mol between the screened compounds and their corresponding target proteins (Figure 2A). Docking simulations predicted potential binding interactions between major bioactive constituents of *Poria cocos* and selected candidate core targets identified from the PPI network, including PI3K, AKT1, TNF-α, IL-6, MAPK3, AGTR1, and MAPK14 (p38) (Figure 2B–I). These predicted interactions were characterized at the atomic level, with several triterpenoid acids showing theoretical hydrogen bond interactions with residues located in the binding regions of targets such as PI3K and MAPK14 (p38). Poricoic acid B was predicted to form two salt bridge interactions with AKT1. Detailed predicted interaction parameters are summarized in Table S1. Overall, the docking results suggested structural plausibility for interactions between major *Poria cocos* components and a subset of PI3K/AKT-associated targets (e.g., PI3K and AKT1) as well as inflammation-related targets, including TNF-α, IL-6, and MAPK14 (p38) (Figure S4A–D). These docking analyses are intended to support exploratory target prioritization rather than to demonstrate functional regulation, causal pathway dependency, or in vivo target engagement, and should be interpreted in conjunction with experimental observations.

### 2.2. In Vivo Validation

#### 2.2.1. *Poria cocos* Extract Reduces LPS/D-GalN-Induced Mortality

**Figure 3:**
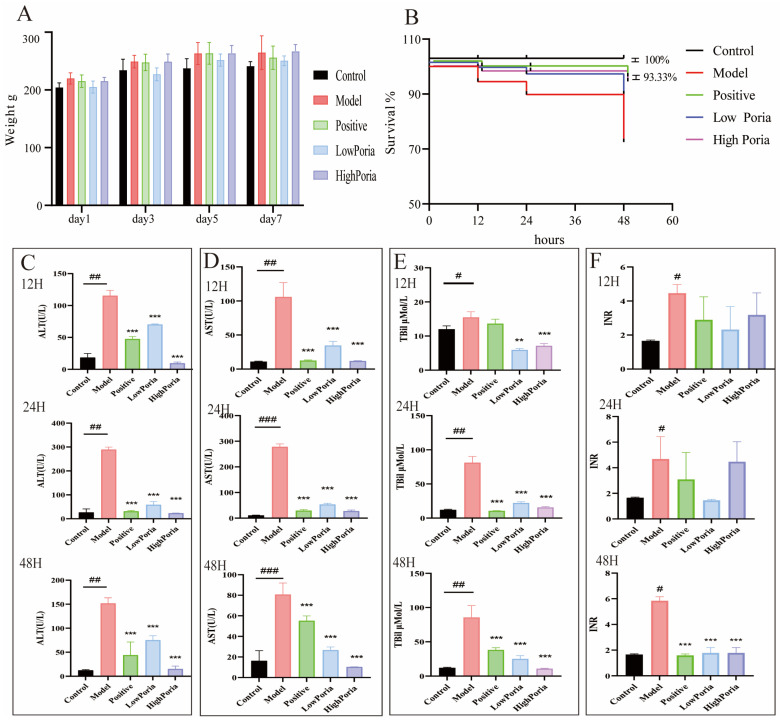
(**A**) Body weight changes in rats. (**B**) Survival curves of rats. (**C**–**E**) Serum levels of alanine aminotransferase ALT, aspartate aminotransferase AST, and total bilirubin (TBil). (**F**) Coagulation function assessed by international normalized ratio (INR). Data are expressed as mean ± SD (*n* = 6). ^#^ *p* < 0.05, ^##^ *p* < 0.01, ^###^ *p* < 0.001 vs. Control group; ** *p* < 0.01, *** *p* < 0.001 vs. Model group.

Body weight was recorded throughout the pretreatment period until ALF induction by LPS/D-GalN to assess the general health status of the rats. As shown in Figure 3A, body weight increased steadily in all groups, and no significant differences were observed between the *Poria cocos* groups and the control or model groups. Survival rates were monitored at 12, 24, and 48 h after LPS/D-GalN administration. Survival was monitored for up to 48 h after LPS/D-GalN administration, and Kaplan–Meier survival curves were generated (Figure 3B). The survival rate of the model group declined progressively, reaching 73.33% at 48 h, whereas the positive group exhibited a higher survival rate of 93.33%. Comparison of survival distributions among groups using the log-rank (Mantel–Cox) test revealed a significant overall difference (*p* = 0.0016). Pretreatment with *Poria cocos* extract was associated with improved survival outcomes. The low-dose *Poria cocos* group showed a survival rate of 93.33% at 24 h, while the high-dose *Poria cocos* group maintained a survival rate of 86.67% at 48 h. These results indicate an association between *Poria cocos* pretreatment and improved survival in the LPS/D-GalN-induced ALF model, based on overall survival curve comparison rather than pairwise post hoc testing.

#### 2.2.2. *Poria cocos* Extract Improves Liver Injury Biomarkers

Combined administration of LPS and D-GalN induced severe liver injury in rats. Serum alanine aminotransferase (ALT) and aspartate aminotransferase (AST) levels were significantly increased at 24 h (289.40 ± 10.39 U/L and 278.60 ± 10.94 U/L, respectively), compared with those in the control group (19.12 ± 7.00 U/L and 12.70 ± 3.10 U/L), with a slight decrease observed at 48 h. Serum total bilirubin (TBil) levels were significantly elevated at both 24 h (81.20 ± 8.97 μmol/L) and 48 h (85.60 ± 17.19 μmol/L), compared with control values (12.01 ± 1.03 μmol/L). International normalized ratio (INR) values peaked at 5.84 ± 0.32 at 48 h, whereas the control group showed an INR of 1.65 ± 0.31.

Pretreatment with *Poria cocos* extract attenuated LPS/D-GalN-induced alterations in liver injury-related biomarkers. Compared with the model group, ALT and AST levels were reduced by 76.73–92.27% and 79.77–91.41%, respectively (*p* < 0.01; Figure 3C,D). Increases in TBil were significantly inhibited, and the high *Poria cocos* group showed near-control levels at 48 h (10.79 ± 0.61 μmol/L; Figure 3E). In addition, coagulation parameters were improved, with INR decreasing to 1.77 ± 0.42 at 48 h (Figure 3F).

#### 2.2.3. Histopathological and Immunohistochemical Evidence of Hepatoprotective Effects

**Figure 4:**
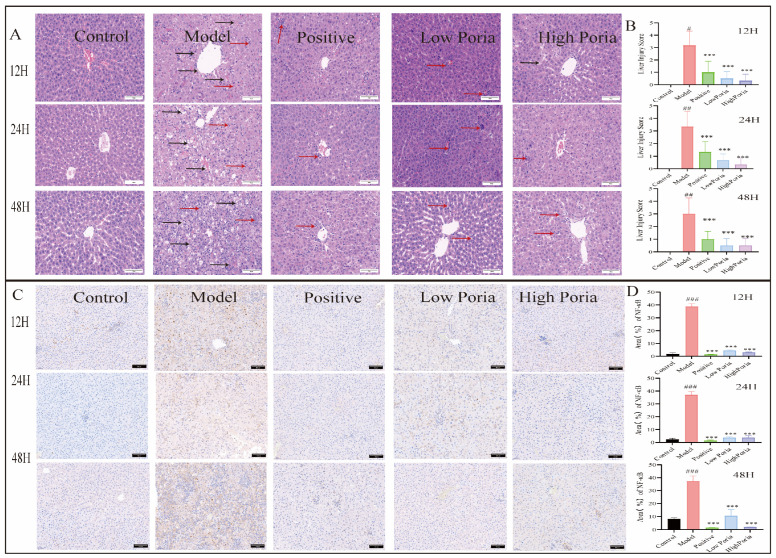
Histopathological and immunohistochemical analysis of liver tissues in different groups. (**A**) Hematoxylin and eosin (H&E) staining of liver tissues (×400 magnification). Black arrows indicate hepatocellular necrosis, and red arrows indicate inflammatory cell infiltration. (**B**) Semi-quantitative histopathological scores of liver injury. (**C**) Immunohistochemical staining of NF-κB in liver tissues (×200 magnification). Brown staining indicates positive NF-κB expression. (**D**) Quantitative analysis of NF-κB-positive staining area. Data are expressed as mean ± SD (*n* = 6). ^#^ *p* < 0.05, ^##^ *p* < 0.01, ^###^ *p* < 0.001 vs. Control group; *** *p* < 0.001 vs. Model group.

Hematoxylin and eosin (H&E) staining showed intact hepatic lobule structures in the control group, whereas the model group exhibited severe structural disruption characterized by hemorrhage, hepatocellular necrosis, and extensive inflammatory cell infiltration. In contrast, liver sections from *Poria cocos*-treated groups, particularly the high *Poria cocos* group, showed attenuated structural disruption compared with the model group (Figure 4A).

A semi-quantitative histopathological analysis was performed on tissue sections collected at 12 h post-induction to assess the extent of liver injury. The model group showed a significantly higher injury score (3.167 ± 1.169) compared with the positive control and *Poria cocos*-treated groups. *Poria cocos* extract reduced histopathological injury scores in a dose-dependent manner (positive control: 1.00 ± 0.89; low *Poria cocos* group: 0.50 ± 0.54; high *Poria cocos* group: 0.16 ± 0.41) (Figure 4B).

Immunohistochemical analysis demonstrated strong NF-κB immunoreactivity in the model group. In contrast, NF-κB immunoreactivity was reduced in the *Poria cocos*-treated groups in a dose-dependent manner (*p* < 0.001; Figure 4C,D).

#### 2.2.4. Effects of *Poria cocos* Extract on Serum IL-6 Levels

**Figure 5:**
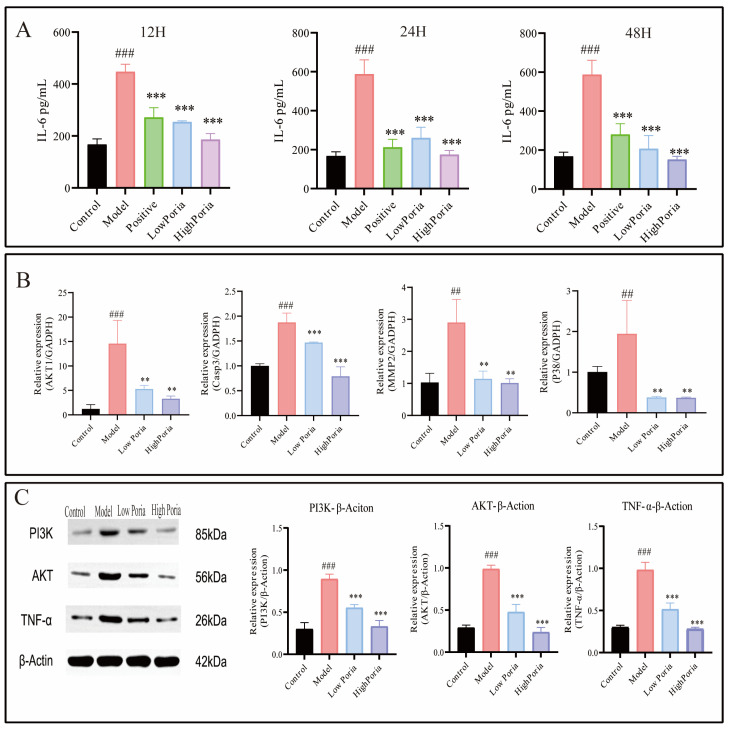
Effects of *Poria cocos* on inflammatory cytokines and key signaling molecules in acute liver failure. (**A**) Serum levels of interleukin-6 (IL-6) determined by enzyme-linked immunosorbent assay (ELISA). (**B**) Relative mRNA expression levels of *AKT*, *Caspase-3*, *MMP2*, and *p38* in liver tissues, measured by quantitative polymerase chain reaction (qPCR). (**C**) Protein expression levels of PI3K, AKT1, and TNF-α in liver tissues, determined by Western blot analysis. Data are expressed as mean ± SD (*n* = 6). ^##^ *p* < 0.01, ^###^ *p* < 0.001 vs. Control group; ** *p* < 0.01, *** *p* < 0.001 vs. Model group.

Enzyme-linked immunosorbent assay (ELISA) results showed that serum IL-6 levels in the model group were significantly elevated at all detected time points (*p* < 0.001). Administration of *Poria cocos* extract significantly reduced serum IL-6 concentration in a time- and dose-dependent manner (Figure 5A).

#### 2.2.5. Effects of *Poria cocos* Extract on PI3K/AKT-Related Gene and Protein Expression

Quantitative polymerase chain reaction (qPCR) analysis performed at 12 h after LPS/D-GalN administration showed that the mRNA expression levels of *AKT1*, *Caspase-3*, *MMP2*, *and MAPK14 (p38)* were significantly increased in the model group compared with the control group (*p* < 0.01). Pretreatment with *Poria cocos* extract markedly attenuated this upregulation in a dose-dependent manner. In the high-dose *Poria cocos* group, the expression levels of *AKT1* (3.30 ± 0.55), *Caspase-3* (0.79 ± 0.19), *MMP2* (1.01 ± 0.13), and *MAPK14 (p38)* (0.37 ± 0.01) were significantly reduced (Figure 5B). Notably, the expression levels of *Caspase-3* and *MMP2* in the high *Poria cocos* group were restored to values comparable to those of the control group (*p* < 0.01).

Consistent with the transcriptional changes, Western blot analysis demonstrated that the protein expression levels of PI3K, AKT1, and TNF-α were markedly elevated in the model group (*p* < 0.001). Pretreatment with *Poria cocos* extract significantly reduced the protein expression levels of PI3K, AKT1, Caspase-3, and p38 in a dose-dependent manner (Figure 5C). Phosphorylation-specific forms of PI3K/AKT were not assessed; therefore, these results reflect changes in total protein abundance rather than functional pathway activation.

Taken together, these results show that pretreatment with *Poria cocos* extract was associated with reduced expression of several inflammation- and apoptosis-related markers, as well as components linked to PI3K/AKT signaling, in the LPS/D-GalN-induced ALF model. The observed decrease in TNF-α protein levels was consistent with attenuation of inflammatory responses detected by immunohistochemical analysis.

## 3. Discussion

*Poria cocos* is a commonly used medicinal fungus in traditional Chinese medicine and has been widely investigated for its bioactive constituents [24]. Chemically, *Poria cocos* is characterized by a triterpenoid-rich profile, predominantly composed of lanostane-type triterpenoids, with minor contributions from sterol derivatives and oleanane-type triterpenoids. Fifteen bioactive components were identified, among which triterpenoids constituted the major fraction, including pachymic acid, dehydrotumulosic acid, and poricoic acids A–C, whereas sterols such as ergosterol and ergosterol peroxide were present in lower abundance. This compositional pattern is consistent with previous phytochemical reports and supports the view that triterpenoids represent the principal bioactive constituents of *Poria cocos* associated with its reported antioxidant, anti-inflammatory, and hepatoprotective properties [25]. Nevertheless, the relevance of these compounds to acute liver failure, particularly under endotoxin-driven conditions, has remained incompletely characterized.

To explore the potential molecular basis of *Poria cocos* in ALF, an integrative strategy combining network pharmacology analysis with in vivo validation was applied. Network analysis identified 178 overlapping targets between *Poria cocos*-related targets and ALF-associated genes, which were mainly enriched in biological processes related to inflammation, cell cycle regulation, and cell migration. KEGG pathway analysis highlighted several inflammation- and survival-related pathways, among which PI3K/AKT-associated signaling appeared repeatedly across enrichment results. This pattern likely reflects the central network position of PI3K/AKT-related components within the predicted compound–target network, rather than pathway dominance. In parallel, molecular docking analysis indicated that multiple triterpenoids exhibited favorable predicted binding affinities toward selected candidate targets, providing a structural basis for prioritizing these compounds and pathways for further investigation. Together, these analyses suggest that *Poria cocos* may influence early-stage ALF through coordinated modulation of multiple interconnected pathways, rather than through a single dominant mechanism.

Protein–protein interaction network analysis further identified a subset of highly connected targets, including PI3K, AKT1, IL-6, TNF-α, MAPK3, MAPK14(p38), and AGTR1, which are known to participate in inflammatory and stress-response signaling relevant to ALF pathophysiology [26]. Docking simulations suggested that representative triterpenoids, such as pachymic acid and poricoic acid derivatives, could adopt stable predicted binding conformations with these targets at the structural level. Although these interactions are based on theoretical modeling and do not demonstrate functional regulation in vivo, they provide a rationale for selecting candidate targets that may warrant further experimental evaluation.

The LPS/D-GalN-induced ALF model used in this study is a well-established experimental system for mimicking endotoxin-driven acute liver injury [20]. Consistent with previous reports, LPS/D-GalN administration resulted in severe liver dysfunction, as reflected by marked elevations in serum ALT, AST, TBil, and INR, accompanied by extensive histopathological damage. Pretreatment with *Poria cocos* extract was associated with improved survival, attenuation of biochemical liver injury markers, and partial preservation of liver histoarchitecture. Because this experimental design focused on pretreatment rather than post-injury intervention, the observed effects primarily reflect a preventive response under conditions of acute inflammatory challenge.

At the molecular level, in vivo experiments showed that *Poria cocos* pretreatment was associated with reduced expression of several inflammation- and apoptosis-related markers, including TNF-α, IL-6, NF-κB, Caspase-3, p38, and MMP2, together with changes in PI3K/AKT-related gene and protein expression. The PI3K/AKT pathway is known to participate in the regulation of inflammatory responses, cell survival, and stress signaling in a context-dependent manner during liver injury [27,28]. In this study, alterations in PI3K/AKT-associated components were consistent with network pharmacology predictions and docking results, suggesting that this pathway may be involved in the observed protective effects. However, without phosphorylation-specific analyses or pathway inhibition experiments, the present data support an associative relationship rather than definitive causal involvement of the PI3K/AKT pathway. Previous studies have reported that PI3K/AKT signaling can exert hepatoprotective effects in acute liver injury models, particularly through limiting excessive inflammatory responses and apoptosis, although its functional role remains highly context-dependent [29,30].

In summary, the results of this study indicate that the protective effects of *Poria cocos* observed in the LPS/D-GalN-induced ALF model are likely related to its triterpenoid-rich chemical composition and multi-target regulatory characteristics. By integrating computational predictions with in vivo observations, this work provides an exploratory framework for understanding how *Poria cocos* may influence acute liver injury. Further studies incorporating pathway-specific validation, post-injury intervention models, and pharmacokinetic analyses will be required to clarify the underlying molecular mechanisms.

## 4. Materials and Methods

### 4.1. Network Pharmacology Analysis

#### 4.1.1. Screening of Bioactive Components and Identification of Disease-Related Targets

Referring to the method of Li et al. [31], bioactive components of *Poria cocos* were retrieved from the Traditional Chinese Medicine Systems Pharmacology Database (TCMSP, https://www.tcmsp-e.com/tcmspsearch.php, accessed on 10 October 2023) and cross-validated via the PubChem database (https://pubchem.ncbi.nlm.nih.gov, accessed on 10 October 2023). Active compounds were screened using predefined criteria of oral bioavailability (OB) ≥ 30% and drug-likeness (DL) ≥ 0.18 [32], which are commonly adopted thresholds in TCMSP-based network pharmacology studies.

Potential protein targets of the selected compounds were predicted using multiple databases to improve the reliability of target identification. Specifically, target information was obtained from TCMSP and the SwissTargetPrediction database (http://www.swisstargetprediction.ch, accessed on 10 October 2023). In addition, PharmMapper (http://www.lilab-ecust.cn/pharmmapper/, accessed on 10 October 2023) was employed for reverse pharmacophore-based target prediction [33], and the Similarity Ensemble Approach (SEA; https://sea.bkslab.org/, accessed on 10 October 2023) database was used for ligand-based target prediction [34]. Targets predicted from all databases were integrated, and duplicate entries were removed. All target identifiers were standardized to official gene symbols using the UniProt database (https://www.uniprot.org, accessed on 10 October 2023), and unannotated entries were excluded.

ALF-related targets were collected from multiple disease-associated databases, including GeneCards (https://www.genecards.org, accessed on 11 October 2023), Online Mendelian Inheritance in Man (OMIM; https://www.omim.org, accessed on 10 October 2023), and DisGeNET (https://www.disgenet.org, accessed on 11 October 2023). Targets retrieved from these databases were merged, and duplicate entries were removed to generate a comprehensive dataset of ALF-related targets for subsequent analyses.

#### 4.1.2. Construction of Drug–Disease Target Network and Functional Enrichment Analysis

Overlapping targets between *Poria cocos*-related targets and ALF-related targets were identified using Venn diagram analysis. These overlapping targets were imported into the STRING database (https://cn.string-db.org/, accessed on 12 October 2023) to construct a protein–protein interaction (PPI) network, with the species limited to Homo sapiens. The resulting PPI network was visualized using Cytoscape (version 3.9.1). Network topological analysis was performed using the CytoNCA plugin to calculate degree, closeness centrality, and betweenness centrality. Based on these topological parameters, highly connected targets were selected for subsequent core network construction. Gene Ontology (GO) functional annotation and Kyoto Encyclopedia of Genes and Genomes (KEGG) pathway enrichment analyses were performed using the DAVID database (https://davidbioinformatics.nih.gov/, accessed on 20 October 2023). Enriched biological processes and signaling pathways were identified based on standard statistical criteria and used for downstream analysis.

#### 4.1.3. Molecular Docking of Bioactive Components with Key Target Proteins

Molecular docking analysis was conducted to evaluate the interaction between bioactive components of *Poria cocos* and selected core target proteins associated with key KEGG pathways. Three-dimensional structures of target proteins were obtained from the Protein Data Bank (PDB). Protein preparation and visualization were performed using PyMOL (Version 3.1.5.1).

Docking simulations were carried out using AutoDock Vina. Ligand structures were prepared in advance, and docking parameters were set according to default protocols. Binding energies were calculated for each ligand–protein complex, and the docking conformation with the lowest predicted binding energy was selected for visualization and further analysis.

### 4.2. Experimental Validation

#### 4.2.1. Preparation of *Poria cocos* Extract

*Poria cocos* was purchased from Yunnan *Poria cocos* Biotechnology Industry Development Co., Ltd. (Kunming, China; Batch No.: Z08230803). Dried *Poria cocos* was crushed and sieved through a 200-mesh sieve, then extracted with 60% ethanol (solid–liquid ratio 1:10, *w*/*v*) at 45 °C for 2 h. Extraction was repeated three times, and the filtrates were combined, concentrated under reduced pressure, and freeze-dried to obtain *Poria cocos* extract.

To provide qualitative compositional characterization of the extract, LC–MS analysis was performed. The analysis was conducted using a liquid chromatography system coupled with a mass spectrometer. Chromatographic separation was achieved on a reversed-phase column using a gradient elution system. Mass spectral data were acquired in full-scan mode, and compound annotation was carried out based on mass-to-charge ratio (m/z) information and database matching. The LC–MS analysis was intended for qualitative identification of representative constituents detectable under the applied analytical conditions, rather than for exhaustive compositional profiling or quantitative determination.

#### 4.2.2. Animal Experiments and Establishment of Acute Liver Failure Model

Male SPF (Specific Pathogen-Free)-grade Sprague-Dawley (SD) rats (7–8 weeks old, body weight 160–180 g) were purchased from Hubei Provincial Center for Experimental Animals (License No.: SCXK (Hubei) 2020–0018). Animals were housed under standard laboratory conditions (12 h light/dark cycle) with free access to food and water. A total of 72 rats were randomly assigned to four groups (n = 18 per group): control group, positive control group (bifendate, 50 mg/kg; purity ≥ 98%, Solarbio, China), low-dose *Poria cocos* extract group (50 mg/kg), and high-dose *Poria cocos* extract group (200 mg/kg) [35]. All treatments were administered once daily by oral gavage for seven consecutive days. Rats in the control group received an equal volume of physiological saline by oral gavage to ensure consistency of handling and experimental procedures across all groups. On day 8, acute liver failure was induced by intraperitoneal injection of lipopolysaccharide (LPS, 50 μg/kg) and D-galactosamine (D-GalN, 800 mg/kg). Rats were euthanized at 12, 24, and 48 h after LPS/D-GalN administration, and blood and liver tissue samples were collected for subsequent analyses. All animal procedures were reviewed and approved by the Animal Ethics Committee of Wuhan Polytechnic University (approval No. WPU202312009) and were conducted in accordance with institutional guidelines for animal care and use, following the principles of Replacement, Reduction, and Refinement (3Rs).

#### 4.2.3. Serum Biochemical Analysis, Histopathological Examination and Immunohistochemistry (IHC)

Blood samples were collected from the abdominal aorta under anesthesia and centrifuged at 3000 rpm for 10 min to separate serum. Serum levels of ALT, AST, TBil, and INR were determined using commercial assay kits from Nanjing Jiancheng Bioengineering Institute (Nanjing, China), while serum IL-6 level was measured with a commercial assay kit purchased from Shanghai Enzyme-linked Biotechnology Co., Ltd. (Shanghai, China), all in strict accordance with the respective manufacturers’ protocols. Liver tissues were fixed in 4% paraformaldehyde, embedded in paraffin, sectioned (4 μm), stained with hematoxylin and eosin (H&E), and examined histopathologically under an optical microscope (Olympus, Tokyo, Japan). The severity of liver injury was evaluated semi-quantitatively by two independent pathologists blinded to the experimental groups, using the Suzuki scoring system (Table 2).

For immunohistochemical analysis, paraffin-embedded liver sections were deparaffinized, rehydrated, and subjected to antigen retrieval. Endogenous peroxidase activity was blocked, followed by incubation with a primary antibody against NF-κB overnight at 4 °C. After incubation with the appropriate secondary antibody, immunoreactivity was visualized using a diaminobenzidine (DAB) substrate and counterstained with hematoxylin. Stained sections were observed and imaged under a light microscope.

#### 4.2.4. Quantitative Real-Time Polymerase Chain Reaction (qPCR) and Western Blot Analysis

Total RNA was extracted from frozen liver tissues using TRIzol reagent (Tiangen, Beijing, China) according to the manufacturer’s instructions. Reverse transcription was performed using a PrimeScript RT reagent kit (Vazyme, Nanjing, China). Quantitative real-time PCR was carried out on a QuantStudio™ 6 Flex Real-Time PCR System (Applied Biosystems, Waltham, MA, USA) using SYBR Green Master Mix (Tolobio, Wuxi, China). Glyceraldehyde-3-phosphate dehydrogenase (GAPDH) was used as the internal reference gene. Relative mRNA expression levels were calculated using the 2^−ΔΔCt^ method. Primer sequences used for qPCR are listed in Table 3.

For Western blot analysis, liver tissues were homogenized in RIPA lysis buffer (Beyotime, Shanghai, China) supplemented with protease and phosphatase inhibitors. Protein concentrations were determined using a bicinchoninic acid (BCA) assay kit (Beyotime, Shanghai, China). Equal amounts of total protein were separated by 10% SDS–polyacrylamide gel electrophoresis and transferred onto polyvinylidene fluoride (PVDF) membranes (Millipore, St. Louis, MO, USA).

Membranes were blocked with 5% non-fat milk for 1 h and incubated overnight at 4 °C with primary antibodies against PI3K, AKT1, TNF-α, and β-actin (Proteintech, Wuhan, China; dilution 1:1000). After washing, membranes were incubated with horseradish peroxidase (HRP)-conjugated secondary antibodies at room temperature for 1 h. Protein bands were visualized using an enhanced chemiluminescence (ECL) detection reagent (Beyotime, Shanghai, China), and band intensities were quantified using ImageJ (Version 1.54) software (NIH, Bethesda, MD, USA).

#### 4.2.5. Statistical Analysis

All data are presented as mean ± standard deviation (SD). Statistical analysis was performed using GraphPad Prism 9.0 (GraphPad Software, San Diego, CA, USA). One-way analysis of variance (ANOVA) followed by Tukey’s post hoc test was used to evaluate differences between groups. A *p* < 0.05 was considered statistically significant.

## 5. Conclusions

In this study, an integrative approach combining network pharmacology analysis and in vivo experiments was used to explore the potential protective effects of *Poria cocos* against LPS/D-GalN-induced acute liver failure. Network pharmacology analysis identified multiple inflammation- and survival-related pathways, including PI3K/AKT-associated signaling, as potential regulatory components. In vivo experiments showed that pretreatment with *Poria cocos* was associated with improved survival, attenuation of liver injury indicators, and reduced expression of several inflammation- and apoptosis-related markers in ALF rats. Changes in PI3K/AKT-related signaling components observed in vivo were consistent with bioinformatics predictions. Taken together, these findings suggest that *Poria cocos* exerts a protective effect in experimental ALF through multi-target and pathway-associated mechanisms. Further studies are required to clarify the precise molecular pathways involved and to inform future exploration of its potential role as an adjunctive preventive strategy for acute liver injury.

## Figures and Tables

**Table 1:** List of Active Compounds of *Poria cocos* with oral bioavailability (OB) ≥ 30% and drug-likeness (DL) ≥ 18% (Selection 409 Criteria).

MolID (Traditional Chinese Medicine Systems Pharmacology Database and Analysis Platform)	Molecule Name	Molecular Weight (MW)	Oral Bioavailability (OB, %)	Drug-Likeness (DL)	PubChem ID
Mol000273	(2R)-2-[(3S,5R,10S,13R,14R,16R,17R)-3,16-dihydroxy-4,4,10,13,14-pentamethyl-2,3,5,6,12,15,16,17-octahydro-1H-cyclopenta[a]phenanthren-17-yl]-6-methylhept-5-enoic acid	470.76	30.93	0.81	10743008
Mol000275	trametenolic acid	456.78	38.71	0.8	12309443
Mol000276	7,9(11)-dehydropachymic acid	526.83	35.11	0.81	15226717
Mol000279	Cerevisterol	430.74	37.96	0.77	10181133
Mol000280	(2R)-2-[(3S,5R,10S,13R,14R,16R,17R)-3,16-dihydroxy-4,4,10,13,14-pentamethyl-2,3,5,6,12,15,16,17-octahydro-1H-cyclopenta[a]phenanthren-17-yl]-5-isopropyl-hex-5-enoic acid	484.79	31.07	0.82	15225964
Mol000282	ergosta-7,22E-dien-3beta-ol	398.74	43.51	0.72	5283628
Mol000283	Ergosterol peroxide	430.74	40.36	0.81	5351516
Mol000285	(2R)-2-[(5R,10S,13R,14R,16R,17R)-16-hydroxy-3-keto-4,4,10,13,14-pentamethyl-1,2,5,6,12,15,16,17-octahydrocyclopenta[a]phenanthren-17-yl]-5-isopropyl-hex-5-enoic acid	482.77	38.26	0.82	9805290
Mol000287	3beta-Hydroxy-24-methylene-8-lanostene-21-oic acid	470.81	38.7	0.81	73402
Mol000289	pachymic acid	528.85	33.63	0.81	5484385
Mol000290	Poricoic acid A	498.77	30.61	0.76	5471851
Mol000291	Poricoic acid B	484.74	30.52	0.75	5471852
Mol000292	poricoic acid C	482.77	38.15	0.75	56668274
Mol000296	hederagenin	414.79	36.91	0.75	73299
Mol000300	dehydroeburicoic acid	453.75	44.17	0.83	15250826

**Table 2:** Standards of Suzuki score of liver injury.

Score	Congestion	Vacuole Degeneration	Necrosis
0	None	None	None
1	Slight	Slight	Single cell
2	Mild	Mild	<30%
3	Moderate	Moderate	31–60%
4	Severe	Severe	>60%

**Table 3:** Primer sequences for qPCR.

Gene	Forward Primer (5′→3′)	Reverse Primer (5′→3′)
*AKT1*	CAAGGACTGCAGGAACGAGT	ACAAGGTGTTCCGAGCTGTT
*MAPK14(p38)*	TCGGCACACTGATGACGAAA	GTCCCCGTCAGACGCATTAT
*MMP2*	GGTGGCAATGGAGATGGACA	CCGGTCATAATCCTCGGTGG
*Caspase-3*	CGGACCTGTGGACCTGAAAA	TAACCGGGTGCGGTAGAGTA
*GAPDH*	GCGAGATCCCGCTAACATCA	CTCGTGGTTCACACCCATCA

## Data Availability

The original contributions presented in this study are included in the article/Supplementary Materials. Further inquiries can be directed to the corresponding authors.

## Supplementary Materials

The following supporting information can be downloaded at: https://www.mdpi.com/article/10.3390/ijms27031403/s1.

## Abbreviations

The following abbreviations are used in this manuscript:

ALF	Acute Liver Failure
ALT	Alanine Aminotransferase
AST	Aspartate Aminotransferase
Tbil	Total Bilirubin
INR	International Normalized Ratio
IL-6	Interleukin-6
TNF-α	Tumor Necrosis Factor-alpha
NF-κB	Nuclear Factor-kappa B
PI3K	Phosphoinositide 3-Kinase
AKT1	Protein Kinase B
PI3K/AKT	Phosphoinositide 3-Kinase/Protein Kinase B
p38	p38 Mitogen-Activated Protein Kinase
MMP2	Matrix Metalloproteinase-2
Caspase-3	Cysteine-aspartic Acid Protease-3
GO	Gene Ontology
KEGG	Kyoto Encyclopedia of Genes and Genomes
LPS	Lipopolysaccharide
D-GalN	D-Galactosamine
qPCR	Quantitative Polymerase Chain Reaction
OB	Oral Bioavailability
DL	Drug-Likeness
PPI	Protein–Protein Interaction
TCMs	Traditional Chinese Medicines
SPF	Specific Pathogen-Free
Control	Control Group
Positive	50 mg/kg Bifendate Group
Low Poria	Low-Dose *Poria cocos* Extract (50 mg/kg) Group
High Poria	High-Dose *Poria cocos* Extract (200 mg/kg) Group
H&E	Hematoxylin and eosin
TCM	Traditional Chinses Medicine
TCMSP	Traditional Chinese Medicine Systems Pharmacology Database and Analysis Platform
LC-MS	Liquid Chromatography-Mass Spectrometry
BP	Biological Process
CC	Cellular Component
MF	Molecular Function
Elisa	Enzyme-Linked Immunosorbent Assay
IHC	Immunohistochemistry
DAB	diaminobenzidine
SD	Sprague-Dawley
