# Supplementary material for: Poria cocos Attenuates LPS/D-Galactosamine-Induced Acute Liver Failure in Rats: An Integrative Exploratory Study Combining Network Pharmacology and In Vivo Validation
Source: Int J Mol Sci. 2026 Jan 30;27(3):1403. doi: 10.3390/ijms27031403 (PMC12897855; doi:10.3390/ijms27031403)

Figure S1. Target prediction workflow

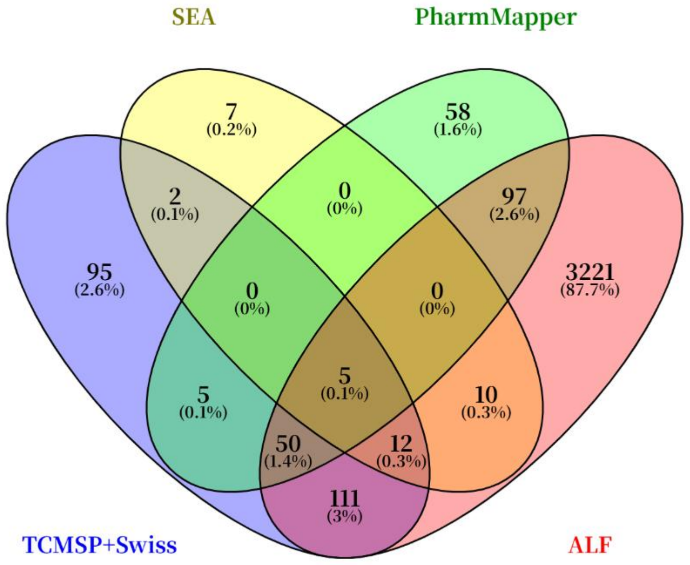

Figure S2. ALF target collection workflow

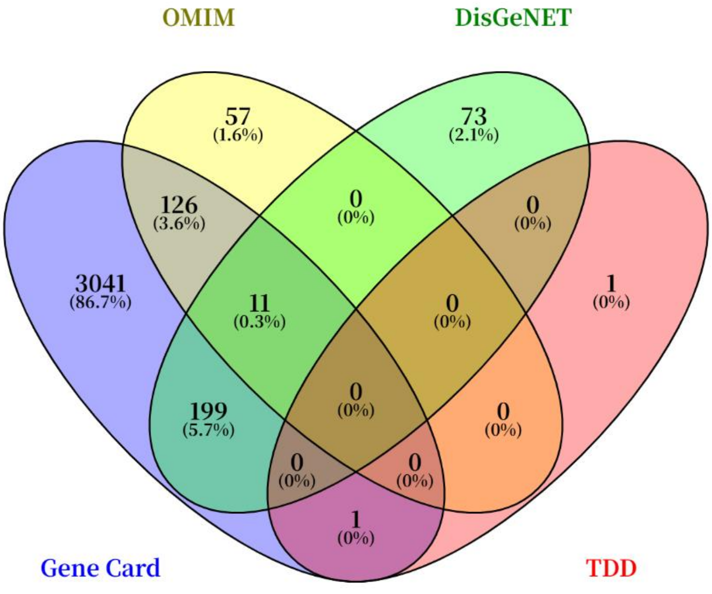

Figure S3. 178 potential targets PPI

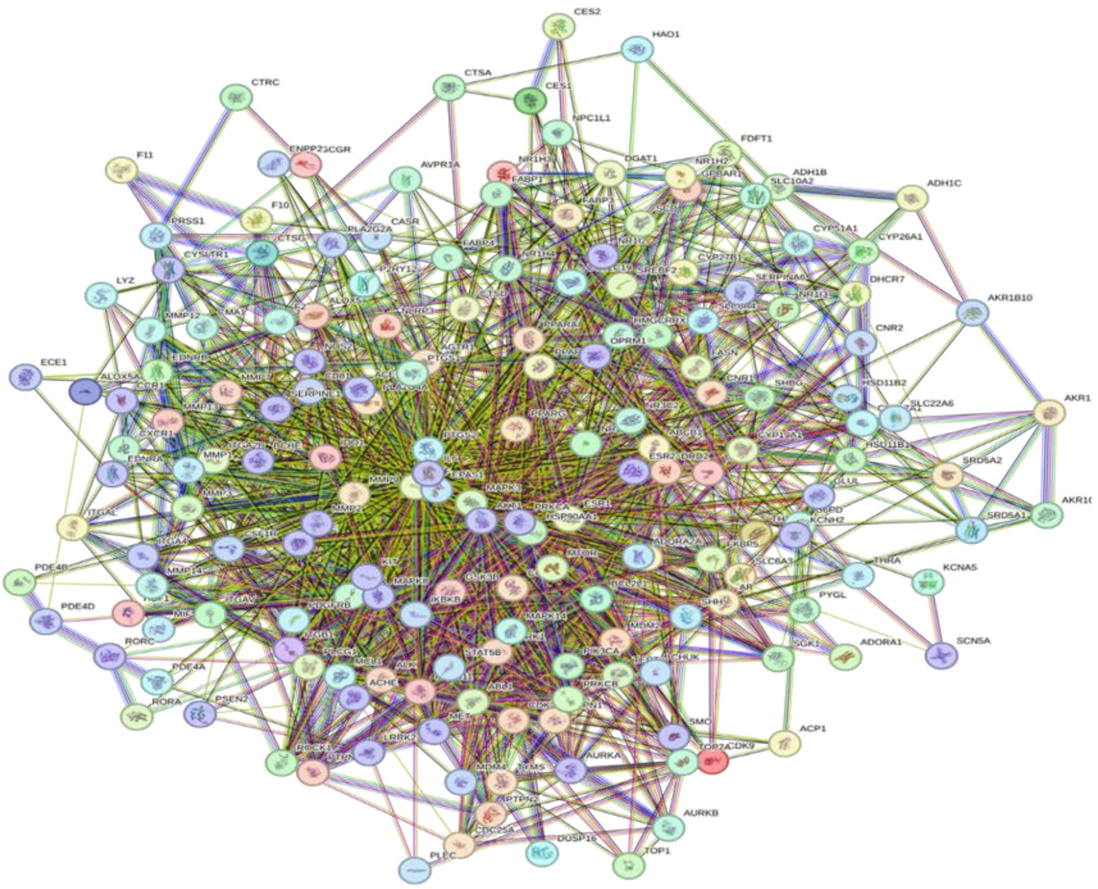

Table S1. Molecular docking results and interaction details of *Poria cocos* bioactive compounds with predicted target proteins.

Ligand-Target Complex	Binding Energy (kcal/mol)	Key Hydrogen Bonds (Residue, Atom, Distance)	Key Hydrophobic Residues[1]	Other Interactions
(2R)-2-[(3S,5R,10S,13R,14R,16R,17R)-3,16-dihydroxy-4,4,10,13,14-pentamethyl-2,3,5,6,12,15,16,17-octahydro-1H-cyclopenta[a]phenanthren-17-yl]-6-methylhept-5-enoic acid - IL-6	-8.1	1. ASP34 (O.co2←O.co2, 2.87 Å)<br>2. LEU178 (O2←O3, 3.80 Å)<br>3. ARG182 (Ng+→O3, 3.59 Å)	ARG30, LEU33, LYS171, GLN175, LEU178, ARG179	The ligand hydroxyl oxygen (O3) acts as a hydrogen bond acceptor for both the LEU178 backbone and ARG182 side chain.
Poricoic acid A - AGTR1	-9.3	1. TYR35 (O3→O.co2, 2.74 Å)<br>2. THR88 (O3→O.co2, 2.82 Å)<br>3. ASP281 (O^-←O.co2, 3.17 Å)	TRP84, TYR87, TYR92, VAL179, ILE288	No

		4. PRO285 (O2←O.co2, 3.22 Å)		
(2R)-2-[(3S,5R,10S,13R,14R,16R,17R)-3,16-dihydroxy-4,4,10,13,14-pentamethyl-2,3,5,6,12,15,16,17-octahydro-1H-cyclopenta[a]phenanthren-17-yl]-6-methylhept-5-enoic acid - PI3K	-7.2	1. ASN65 (Nam→O.co2, 3.20 Å)<br>2. TYR66 (O2←O.co2, 3.03 Å)	TYR69, ALA10, ASN65	No
Cerevisterol - TNF-α	-7.1	1. LYS65 (N3+→O3, 3.42 Å)<br>2. GLN67 (Nam→O3, 3.09 Å)<br>3. ASP140 (O2←O3, 3.48 Å)	PRO20, GLU23	No
3beta-Hydroxy-24-methylene-8-lanostene-21-oic acid - MAPK3	-7.6	1. GLU94 (O^-←O3, 3.58 Å)<br>2. ALA172 (Nam→O.co2, 3.38 Å)<br>3. ALA172 (O2←O.co2, 2.79 Å)	PHE350, LEU351, TRP352, ARG90	No
(2R)-2-[(3S,5R,10S,13R,14R,16R,17R)-3,16-dihydroxy-4,4,10,13,14-pentamethyl-2,3,5,6,12,15,16,17-octahydro-1H-cyclopenta[a]phenanthren-17-yl]-5-isopropyl-hex-5-enoic acid - MAPK14	-9.4	1. LYS53 (N3+→O3, 2.85 Å)<br>2. GLU71 (O.co2←O3, 2.70 Å)<br>3. MET109 (Nam→O3, 4.02 Å)<br>4. MET109 (O2←O3, 2.48 Å)	VAL30, VAL38, LEU167, ILE84, LEU75	No
Cerevisterol - AKT1 (Site 1)	-8.3	1. GLU837 (O.co2←O3, 3.92 Å)	ILE672, PHE673, VAL676, PRO723	No
Poricoic acid B - AKT1 (Site 2)	-8.3	1. TRP22 (N3→O3, 3.21 Å)2. GLU829 (O^-←O.co2, 3.06 Å)3. ASN833	THR21, ARG69, LEU830	Salt bridges: Forms two salt bridges with ARG15 (4.46 Å) and ARG840 (4.66 Å).

Table notes: Key hydrophobic residues refer to major non-polar contact sites with ligand atoms at distances < 4.0 Å. The arrow "→" indicates hydrogen bond direction from donor to acceptor. Atom labels include O.co2 (carboxyl oxygen), O^- (deprotonated carboxyl oxygen), Nam (backbone amide nitrogen), O3 (ligand hydroxyl oxygen), and N3 (indole nitrogen). Ligands were tentatively identified and docking interactions represent theoretical predictions.

Figure S4A. Predicted binding mode of Poricoic acid B within the AKT1 binding pocket. Poricoic acid B (orange) is shown interacting with AKT1 residues (blue). Hydrogen bonds are indicated by blue dashed lines, and salt-bridge interactions are highlighted by yellow dashed lines. This complex exhibits multiple stabilizing interactions, including salt bridges with key arginine residues, consistent with the docking results summarized in Table S1.

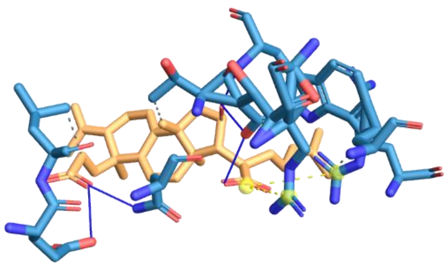

Figure S4B. Predicted binding mode of a representative triterpenoid component of Poria cocos within the PI3K binding pocket. The ligand (orange) and surrounding PI3K residues (blue) are shown. Predicted hydrogen-bond and non-covalent interactions are indicated by dashed lines. This diagram complements the docking results summarized in Table S1.

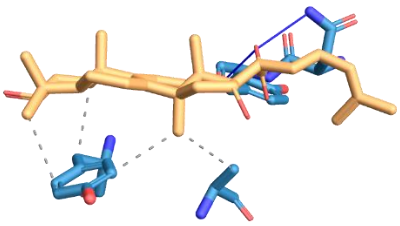

Figure S4C. Predicted binding mode of a representative Poria cocos triterpenoid within the MAPK14 (p38) binding pocket. The ligand (orange) and surrounding MAPK14 residues (blue) are shown. Predicted hydrogen-bond and hydrophobic interactions are indicated by dashed lines. This docking pose is consistent with the interaction details summarized in Table S1.

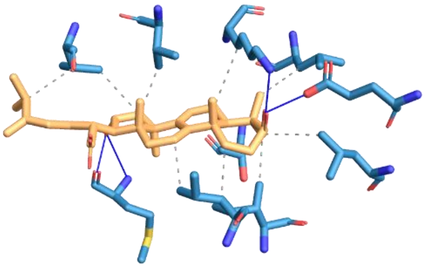

Figure S4D. Predicted binding mode of Poricoic acid A within the AGTR1 binding pocket. The ligand (orange) and surrounding AGTR1 residues (blue) are shown. Predicted non-covalent interactions are indicated. This diagram is provided to illustrate a representative ligand–target interaction supporting the multi-target characteristics of Poria cocos components, as summarized in Table S1.

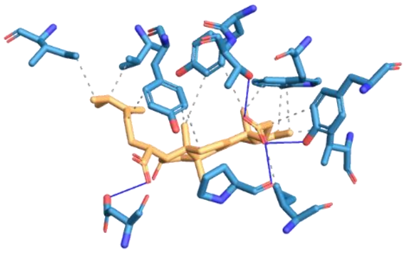

Figure S5. Representative LC–MS chromatogram of Poria cocos extract.

Peaks corresponding to several characteristic triterpenoids are indicated, with tentative identifications based on mass-to-charge ratio (m/z) and comparison with literature data: 1. (2R)-2-[(3S,5R,10S,13R,14R,16R,17R)-3,16-dihydroxy-4,4,10,13,14-pentamethyl-2,3,5,6,12,15,16,17-octahydro-1H-cyclopenta[a]phenanthren-17-yl]-6-methylhept-5-enoic acid; 2. trametenolic acid; 3. (2R)-2-[(3S,5R,10S,13R,14R,16R,17R)-3,16-dihydroxy-4,4,10,13,14-pentamethyl-2,3,5,6,12,15,16,17-octahydro-1H-cyclopenta[a]phenanthren-17-yl]-5-isopropyl-hex-5-enoic acid; 4. Poricoic acid A; 5. Poricoic acid B. (Note: Identifications are preliminary, based on accurate mass and retention time alignment with reported values for Poria cocos constituents; confirmation with authentic standards was not performed in this study.)

This analysis was performed for qualitative compositional characterization to confirm the presence of triterpenoid-related constituents, rather than for comprehensive profiling or quantitative determination.

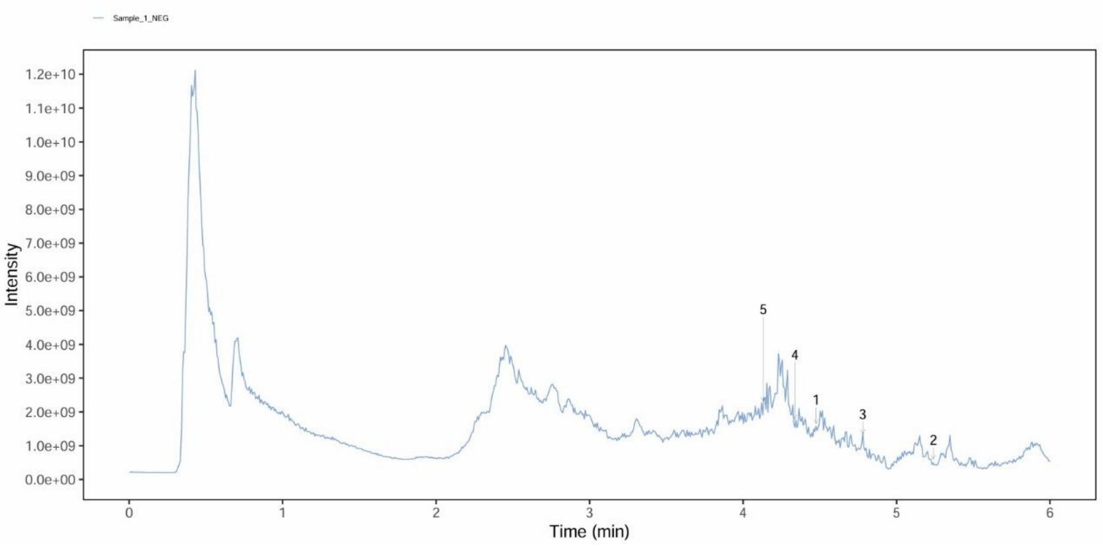

Supplement: Supplementary file 1 [file ijms-27-01403-s001.zip › ijms-4099609-supplementary.pdf]
